# Increased circulating VCAM-1 correlates with advanced disease and poor survival in patients with multiple myeloma: reduction by post-bortezomib and lenalidomide treatment

**DOI:** 10.1038/bcj.2016.37

**Published:** 2016-05-27

**Authors:** E Terpos, M Migkou, D Christoulas, M Gavriatopoulou, E Eleutherakis-Papaiakovou, N Kanellias, M Iakovaki, I Panagiotidis, D C Ziogas, D Fotiou, E Kastritis, M A Dimopoulos

**Affiliations:** 1 Department of Clinical Therapeutics, National and Kapodistrian University of Athens, School of Medicine, Athens, Greece

## Abstract

Circulating vascular cell adhesion molecule-1 (VCAM-1), intercellular adhesion molecule-1 (ICAM-1) and selectins were prospectively measured in 145 newly-diagnosed patients with symptomatic myeloma (NDMM), 61 patients with asymptomatic/smoldering myeloma (SMM), 47 with monoclonal gammopathy of undetermined significance (MGUS) and 87 multiple myeloma (MM) patients at first relapse who received lenalidomide- or bortezomib-based treatment (RD, *n*=47; or VD, *n*=40). Patients with NDMM had increased VCAM-1 and ICAM-1 compared with MGUS and SMM patients. Elevated VCAM-1 correlated with ISS-3 and was independently associated with inferior overall survival (OS) (45 months for patients with VCAM-1 >median vs 75 months, *P*=0.001). MM patients at first relapse had increased levels of ICAM-1 and L-selectin, even compared with NDMM patients and had increased levels of VCAM-1 compared with MGUS and SMM. Both VD and RD reduced dramatically serum VCAM-1 after four cycles of therapy, but only VD reduced serum ICAM-1, irrespective of response to therapy. The reduction of VCAM-1 was more pronounced after RD than after VD. Our study provides evidence for the prognostic value of VCAM-1 in myeloma patients, suggesting that VCAM-1 could be a suitable target for the development of anti-myeloma therapies. Furthermore, the reduction of VCAM-1 and ICAM-1 by RD and VD supports the inhibitory effect of these drugs on the adhesion of MM cells to stromal cells.

## Introduction

Multiple myeloma (MM) cell growth and survival is regulated by interactions between myeloma and bone marrow stromal cells.^1^ The adherence of myeloma cells to stromal cells and to components of the extracellular matrix through adhesion molecules is a key process for this regulation.^2^ These adhesion molecules include vascular cell adhesion molecule-1 (VCAM-1; CD106), intercellular adhesion molecule-1 (ICAM-1; CD54), CD44, CD58, selectins and others.^3^ VCAM-1 is a member of the immunoglobulin superfamily and encodes a cell surface sialoglycoprotein. VCAM-1 protein is an endothelial ligand for very late antigen-4 (VLA-4 or α4β1) of the β1 subfamily of integrins and is implicated in the homing and migration of malignant cells, including MM cells.^4, 5^ ICAM-1 is an intracellular adhesion molecule expressed on the membrane of leukocytes and on endothelial cells. It is the ligand for leukocyte function-associated antigen 1; the interaction of ICAM-1 with its ligand on the surface of MM cells leads to MM cell proliferation in mouse models^6^ and it is involved in drug resistance of myeloma cells.^7^ Antibodies against ICAM-1 have shown anti-myeloma activities *in vitro* and *in vivo*,^6^ while a phase 1 study with an anti-ICAM-1 monoclonal antibody has shown encouraging results in patients with relapsed/refractory MM.^8^

Finally, selectins also have an important role in the biology of MM. Activated platelets express P-selectin, the largest of the selectin molecule family, which is responsible for early leukocyte recruitment during the inflammatory response.^9^ L-selectin is a small intravascular molecule, which is mainly expressed by leukocytes;^10^ and E-selectin is expressed on the activated endothelium during inflammation.^11^ In MM, it has been shown that P-selectin glycoprotein ligand 1 is overexpressed on myeloma cells and regulates the adherence and homing of myeloma cells in the bone stroma through its interaction with selectins.^12^ Furthermore, interactions between P-selectin with its ligand offer macrophage-induced drug resistance for myeloma cells.^7^

Although the role of adhesion molecules has been established in the biology of MM, there is limited information in the literature for the correlation of circulating levels of different adhesion molecules with disease features and prognosis of MM patients. Thus, the aim of this prospective study was to evaluate the circulating levels of VCAM-1, ICAM-1, P-, L- and E-selectin in myeloma patients, to explore possible correlation with disease characteristics, including survival and to investigate the effect of anti-myeloma agents, such as bortezomib and lenalidomide, on their levels.

## Patients and methods

### Study design

This was a prospective study for the evaluation of circulating levels of adhesion molecules in myeloma patients, their correlation with features of the disease, including survival and possible alterations after anti-myeloma therapy with bortezomib or lenalidomide plus dexamethasone therapy.

#### Inclusion and exclusion criteria

The inclusion criteria of the study included (i) adult patients with newly-diagnosed symptomatic myeloma (NDMM) before the administration of any kind of therapy; (ii) myeloma patients at the time of their first relapse (RMM) who are treated with bortezomib or lenalidomide plus dexamethasone therapy; (iii) patients who have given their written informed consent for blood sampling and for recording of their medical data which is pertinent to the purposes of this study.

The exclusion criteria included (i) patients <18 years; (ii) presence of heart disease (cardiac failure or angina) or hypertension that could alter the measurement of the studied circulating molecules; (iii) presence of autoimmune disorders; (iv) use of medication that could alter the levels of these parameters (that is, anti-hypertension drugs, aspirin or other drugs with anti-platelet activity, anti-coagulants) during the last 6 months before measurement.

#### Study end points

The primary end point of the study was the evaluation of circulating levels of adhesion molecules (VCAM-1, ICAM-1, P-, L- and E-selectin) in NDMM at the time of diagnosis and their comparison with those of patients with monoclonal gammopathy of undetermined significance (MGUS) and asymptomatic/smoldering myeloma (SMM).

Secondary end points included (i) correlation of circulating levels of adhesion molecules with disease features (stage, bone disease, LDH, plasma cell infiltration and so on); (ii) correlation of adhesion molecules with overall survival (OS) of NDMM; (iii) evaluation of circulating adhesion molecules in RMM at the time of first relapse; (iv) alterations of the levels of adhesion molecules after the administration of four cycles of bortezomib plus dexamethasone (VD) or after the administration of four cycles of lenalidomide plus dexamethasone (RD) in RMM patients treated at first relapse.

#### Patients' enrolment

The enrolment period was between January 2008 and January 2011. Patients were informed of the objectives and the details of the study before giving their approval and signing the informed consent forms. The study was conducted according to the principles defined by the 18th World Medical Association Assembly (Declaration of Helsinki, 1964) and all its future amendments. The study protocol was designed and executed according to the guidelines and regulations pertaining to studies in Greece as well as the Good Clinical Practice Guidelines as defined by the International Conference of Harmonization. The study was approved by the local ethics committee.

#### Control groups

In this study, circulating levels of VCAM-1, ICAM-1, P-, L- and E-selectin were also measured in 47 patients with MGUS (22M/25F, median age 70 years) and 61 patients with SMM (27M/34F, median age 63 years) at the time of their diagnosis. MGUS and SMM patients were diagnosed during the same recruitment period. The medical history of MGUS and SMM patients was recorded in order to assure that they had no history of cardiovascular or autoimmune disorder and did not receive any drug that could alter adhesion molecules during the last 6 months (i.e. anti-hypertension drugs, aspirin or other drugs with anti-platelet activity or anti-coagulants).

#### Data recording and quality assurance

Data were collected from the medical files of the enrolled patients. Clinical study monitoring was performed for source data verification and ensured the accuracy of the data. Treatment data, treatment outcome according to IMWG criteria^13^ and OS were also recorded.

### Statistical analysis

Differences between patients and controls as well as between different patient subsets were evaluated using the Mann–Whitney test. Differences between baseline and 4-cycle values of the studied parameters post-RD or post-VD were evaluated using the Wilcoxon signed-rank test. The Spearman Rank correlation test was employed to examine relationships between various parameters and clinical patient characteristics. Logarithmic transformation was also used for heavily skewed variables. Survival was calculated from the day of treatment initiation until the date of death or last follow-up. Patients who were lost to follow-up were censored at the date of last contact. Survival probabilities were calculated by the Kaplan–Meier method and comparisons made using the log-rank test to identify the potential prognostic factors. Variables found to be statistically significant at the *P<*0.05 level were entered into a multivariate model using Cox regression analysis to identify the most statistically significant model. All *P-*values were two sided, the level of significance was <0.05 and confidence intervals refer to 95% boundaries. All analyses were performed using SPSS 20 software (IBM SPSS statistics for Windows, Version 20.0, IBM Corp, Armonk, NY, USA).

### Measurement of circulating adhesion molecules

After vein puncture, serum was separated within 4 h and stored at −80 °C for all newly-diagnosed myeloma patients and controls. For MM patients at first relapse, serum was obtained on day 1 of the first RD or VD cycle and then on day 28 of the fourth RD cycle or on day 21 of the fourth VD cycle and was stored at −80 °C, until the date of measurement. An enzyme-linked immunosorbent assay (ELISA) was used for the detection of VCAM-1, ICAM-1, P-, L- and E-selectin (R&D Systems, Minneapolis, MN, USA), according to the manufacturer's instructions. Measurements were performed approximately every 3 months; this was the longer period that a serum sample was stored.

## Results

### Patients

One hundred and forty-five NDMM patients were studied. The characteristics of MM patients and control groups are shown in Table 1. All symptomatic patients received frontline treatment with novel agent-based regimens (thalidomide-, lenalidomide- (only 17 patients) or bortezomib-based therapies).

Eighty-seven RMM patients at first relapse were also evaluated prospectively. Forty-seven patients received RD. The standard dose of lenalidomide, that is, 25 mg PO daily, on days 1–21 of a 28-day cycle, was given to patients with a baseline creatinine clearance of >50 ml/min. For patients with lower creatinine clearance adjustments of lenalidomide were made, according to guidelines.^14^ Dexamethasone was administered at a dose of 40 mg PO on days 1–4 and 15–18 for the first four cycles and on days 1–4 thereafter. Forty patients received VD. Bortezomib was administered at the standard dose of 1.3 mg/m^2^, intravenously, on days 1, 4, 8 and 11 of a 21- day cycle, while dexamethasone was given at a dose of 20 mg/m^2^ per os, on days 1–2, 4–5, 8–9 and 11–12.

### Circulating levels of adhesion molecules in newly-diagnosed myeloma patients and controls

**Figure 1:**
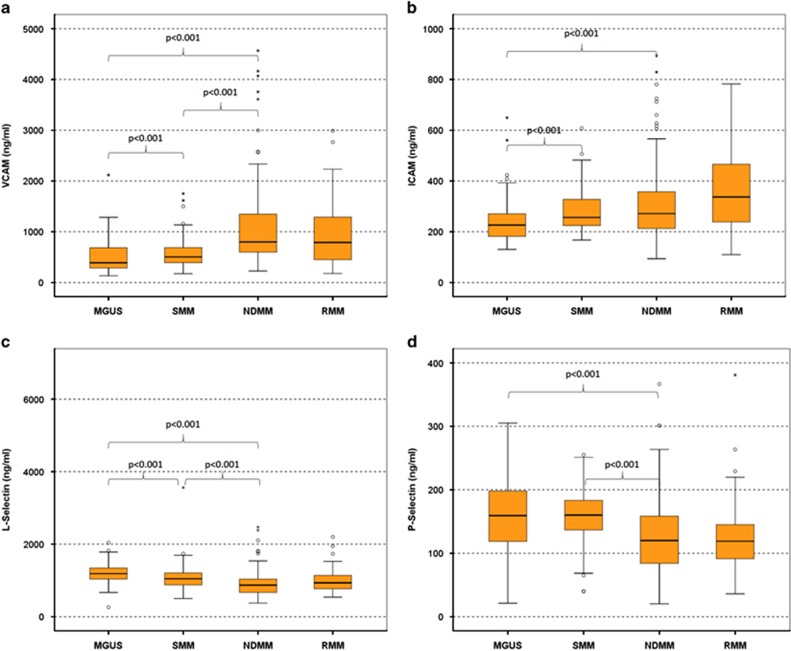
Circulating levels of VCAM (**a**), ICAM (**b**), L-selectin (**c**) and P-selectin (**d**) in all patients' groups; whisker plots (open circles) refer to outlier values, while 'extreme' values are marked with an asterisk.

Patients with NDMM had increased levels of VCAM-1 (median (range): 799 ng/ml (226–4567 ng/ml); Figure 1a) and ICAM-1 (271 ng/ml (93–894 ng/ml); Figure 1b) compared with MGUS patients (388 ng/ml (131–2117 ng/ml) and 226 ng/ml (130–649 ng/ml), respectively (*P<*0.01 for both comparisons)), but only VCAM-1 was increased compared with SMM patients (504 ng/ml (174–1751 ng/ml), *P<*0.001) (Table 2). Patients with SMM had increased VCAM-1 and ICAM-1 levels and decreased L-selectin levels compared with MGUS patients (Figures 1a–c). Patients with NDMM had also decreased levels of both L- and P-selectin (median (range): 870 ng/ml (374–2473 ng/ml) and 120 ng/ml (20–366 ng/ml), respectively) compared with SMM (1046 ng/ml (860–1224 ng/ml) and 160 ng/ml (40–255 ng/ml), respectively (*P<*0.001 for both comparisons; Figures 1c and d)) as well as compared with MGUS patients (1187 ng/ml (262–2039 ng/ml) and 159 ng/ml (21–305 ng/ml), respectively (*P<*0.001 for both comparisons)). There were no differences between the three groups regarding E-selectin levels.

**Figure 2:**
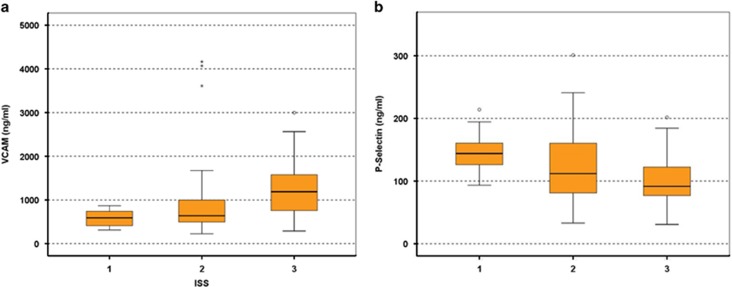
Correlation of VCAM (**a**) and P-selectin (**b**) with ISS stage: VCAM-1 was lower in NDMM patients with ISS-1 compared to patients with ISS-2 and ISS-3 (ANOVA *P*=0.002; **a**); P-selectin was higher in ISS-1 compared with ISS-2 and ISS-3 NDMM (ANOVA *P*=0.001; **b**); whisker plots (open circles) refer to outlier values, while 'extreme' values are marked with an asterisk.

VCAM-1 was lower in NDMM patients with ISS-1 (590 ng/ml (312–869 ng/ml)) compared with patients with ISS-2 (639 ng/ml (226–4163 ng/ml)) and ISS-3 (1189 ng/ml (288–2997 ng/ml), ANOVA *P*=0.002; Figure 2a), while P-selectin was higher in ISS-1 (143 ng/ml (93–214 ng/ml)) compared with ISS-2 (111 ng/ml (33–301 ng/ml)) and ISS-3 NDMM (91 ng/ml (30–201 ng/ml), ANOVA *P*=0.001; Figure 2b).

For all newly-diagnosed patients, there was a strong correlation between VCAM-1 and ICAM-1 (*r*=0.466, *P<*0.001) and a negative correlation between VCAM-1 and P-selectin (*r*=−0.216, *P<*0.001). Serum VCAM-1 showed strong positive correlations with β2-microglobulin (*r*=0.560, *P<*0.001), urea (*r*=0.430, *P<*0.001) and creatinine (*r*=0.436, *P<*0.001) and negative correlation with albumin (*r*=−0.167, *P*=0.018). P- and L-selectin selectin negatively correlated with β2-microglobulin (*r*=−0.430 and *r*=−0.215 respectively; *P<*0.001 for both), creatinine (*r*=−0.339 and *r*=−0.284; *P<*0.001) and urea (*r*=−0.321 and *r*=−0.251; *P<*0.001), and positively correlated with albumin (*r*=0.354 and *r*=0.381, *P<*0.001). There were no correlations for E-selectin and ICAM-1 with β2-microglobulin, creatinine, urea or albumin.

**Figure 3:**
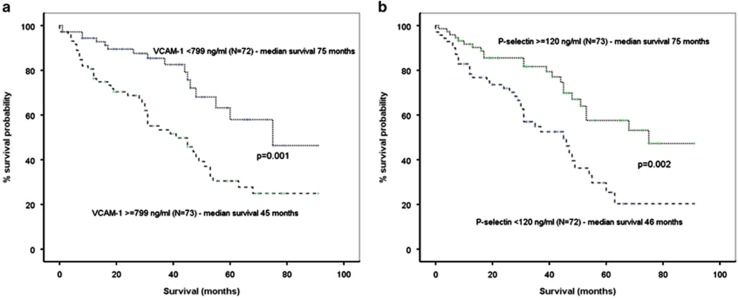
High levels of VCAM and low levels of P-selectin correlate with shorter survival: patients with VCAM levels above median value had a median OS of 45 months in comparison with 75 months of all others (*P*=0.001; **a**), while patients with low P-selectin (below median) had a median OS of 46 months compared with 75 months of all others (*P*=0.002; **b**).

The median follow-up of patients with NDMM was 51 months and the median OS was 53 months (CI 95% 46–59 months). Increased levels of VCAM-1 (>median) and decreased levels of P-selectin (<median) were predicted for inferior survival. In particular, patients with VCAM-1 >median had a median OS of 45 months in comparison with 75 months of all others (*P*=0.001; Figure 3a), while patients with low P-selectin (below median) had a median OS of 46 months compared with 75 months of all others (*P*=0.002; Figure 3b). Both VCAM-1 and P-selectin as continuous variables were also prognostic for survival of NDMM patients (*P*=0.003 and *P*=0.017, respectively). Other important factors for survival in the univariate analysis included ISS stage (*P*=0.026) and LDH >300 U/l (*P<*0.001). In the multivariate analysis that included the above factors, VCAM-1<median (HR: 0.444 (CI 95%: 0.246–0.801); *P*=0.007), LDH<300 U/l (HR: 0.350 (CI 95%: 0.133–0.919); *P*=0.033) and ISS (HR: 0.392 (CI 95%: 0.225–0.681); *P*=0.001) correlated independently with survival.

### Circulating levels of adhesion molecules in myeloma patients at first relapse

**Figure 4:**
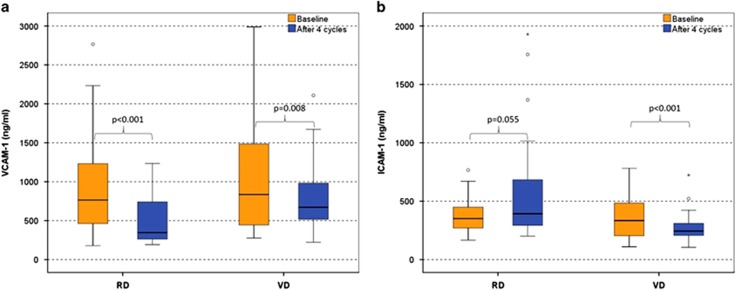
Alterations of VCAM (**a**) and ICAM (**b**) post four cycles of RD and VD; whisker plots (open circles) refer to outlier values, while 'extreme' values are marked with an asterisk.

**Figure 5:**
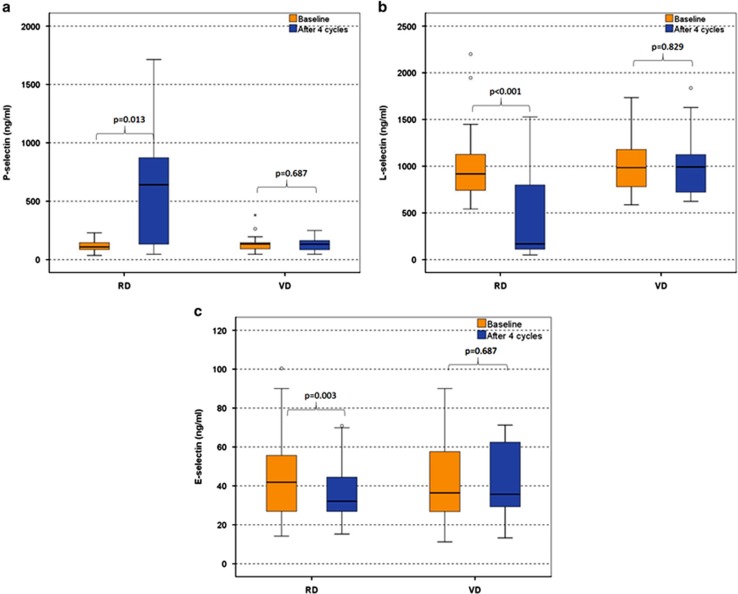
Alterations of P-selectin (**a**), L-selectin (**b**) and E-selectin (**c**) post four cycles of RD and VD; whisker plots (open circles) refer to outlier values, while 'extreme' values are marked with an asterisk.

RMM patients at first relapse had increased levels of ICAM-1 and L-selectin compared with NDMM patients (*P*=0.001 and *P*=0.017, respectively) and increased levels of VCAM-1 and decreased levels of P-selectin compared with SMM and MGUS patients (*P<*0.01 for all comparisons; Table 2). VCAM-1 levels were similar for patients with NDMM and RMM. Both VD and RD administration reduced dramatically serum levels of VCAM-1 after four cycles of therapy (*P<*0.001 and *P*=0.008 respectively; Figure 4a). The reduction of VCAM-1 was more pronounced with RD than with VD (median % reduction: −38 vs −17% *P*=0.002; Figure 4a). However, only VD reduced serum levels of ICAM-1 (*P*=0.001; Figure 4b). Therapy with RD increased P-selectin and decreased L- and E-selectin (*P*⩽0.01 for all comparisons; Figures 5a–c), while VD had no effect on serum levels of selectins. All the changes in the levels of the adhesion molecules occurred irrespective of response to therapy.

## Discussion

Adhesion molecules such as VCAM-1, ICAM-1 and selectins mediate the homing of MM cells to the BM and are implicated in the MM cell growth and survival.^1, 2, 3, 15^ There is very limited information in the literature regarding the circulating levels of adhesion molecules in myeloma patients and their significance. In our study, we evaluated a large number of myeloma patients and found that patients with NDMM had higher levels of VCAM-1 and ICAM-1 compared with MGUS patients and higher VCAM-1 levels compared with SMM patients. Furthermore, high VCAM-1 circulating levels correlated with advanced ISS stage and inferior survival in both the univariate and multivariate analysis in newly-diagnosed myeloma patients. Importantly, although the levels of VCAM-1 correlated with features of tumor burden (such as β2-microblobulin), the prognostic significance of serum VCAM-1 was independent of ISS stage.

Elevated circulating levels of VCAM-1 and ICAM-1 have been also described in two small studies, where E-selectin has shown confounding results.^16, 17^ In our study, the levels of L- and P-selectin were decreased in patients with NDMM in comparison with SMM and MGUS, but there was no difference in the levels of E-selectin for the three studied patients' groups. Different populations, different number of patients, variability in the circulating levels of adhesion molecules and different methodology may explain such differences among studies.

Our results also suggest that different adhesion molecules have a unique pattern of circulating levels between patients with MGUS, SMM or MM. For VCAM-1 and ICAM-1, the levels of the soluble form increase from MGUS to SMM and to NDMM. On the other hand, we observed that the levels of soluble L- and P-selectin were decreased from MGUS to SMM and MM. Patients at relapse also had increased levels of VCAM-1 and ICAM-1 compared with SMM and MGUS, confirming the pattern that was observed in NDMM patients. These data suggest that more advanced disease correlates with higher VCAM-1 and possibly with lower selectin levels, mainly lower P-selectin. Indeed, patients with ISS-3 myeloma at diagnosis had higher levels of VCAM-1 compared with ISS-1 and ISS-2. These findings only party explain the correlation of VCAM-1 with patients' survival, indicating additional roles and mechanisms for adhesion molecules, and mainly of VCAM-1 in MM. It is the first time where circulating levels of an adhesion molecule independently correlate with survival in myeloma patients and this confirms preclinical findings where VCAM-1 seems to have an important role in the myeloma cell growth and progression.^5, 18^ Especially, in the B9/BM1 syngeneic murine bone marrow myeloma model, the myeloma cell growth was based on VCAM-1 and was completely suppressed by an anti-VCAM-1 monoclonal antibody. Furthermore, VCAM-1 as well as other adhesion molecules, such as ICAM-1, are involved in a mechanism called 'cell adhesion-mediated drug resistance' (CAM-DR), which is thought to be one of the major mechanisms by which MM cells escape the cytotoxic effects of therapeutic agents.^7, 19^ Primarily, multidrug-resistant patients were shown to have significantly higher expression levels of VLA-4 (the receptor of VCAM-1) and ICAM-1 than responders, suggesting that these molecules are implicated in chemotherapeutic agent resistance by myeloma cells.^20^

In patients at first relapse, both RD and VD reduced the levels of VCAM-1, although this reduction was more pronounced with RD. On the other hand, levels of ICAM-1 were reduced only by VD, and levels of P-selectin were increased after treatment with RD. Our observations indicate that both agents could overcome, at least partially, the drug resistance of MM, which is due to these adhesion molecules. Also, the fact that these reductions were independent of the response to therapy indicates that these drugs also act on other sites of the microenvironment beyond the plasma cell. Indeed, bortezomib was found to overcome CAM-DR through the suppression of VLA-4 expression in preclinical studies.^21^ Furthermore, it has been shown that both bortezomib and lenalidomide exert their anti-myeloma properties by directly killing the myeloma cells and indirectly through different actions, including the ability to inhibit adherence of myeloma cells to stromal cells.^3^

In conclusion, our study suggests that patients with newly-diagnosed MM have increased serum levels of VCAM-1 and ICAM-1 that correlate with advanced disease features. VCAM-1 is also independently correlated with survival, suggesting an important role in the biology of the disease and supporting its targeting with novel anti-myeloma drugs. The administration of bortezomib and lenalidomide resulted in the reduction of VCAM-1 and ICAM-1 and supports an inhibitory effect of these drugs on the adhesion of MM cells to stromal cells.

## Figures and Tables

**Table 1:** Patients characteristics

	*Newly-diagnosed symptomatic MM*	*Newly-diagnosed asymptomatic MM*	*Relapsed MM (first relapse)*	*MGUS*
Number of patients	145	61	87	47
Gender (M/F)	68/77	27/34	42/45	22/25
Age, median (range)	72 (36–91)	63 (33–86)	71 (40–85)	70 (57–80)
Type of MM: IgG/IgA/BJ/NS	82/36/17/10	42/17/2/0	55/21/11/0	36/10/1/0
Stage at diagnosis (ISS) I/II/III	33/45/67	55/6/0	23/38/26	
				
*Parameters at baseline*				
Hb <10 g/dl	104	0	79	0
Creatinine >upper normal limit	45	0	36	1
Creatinine >2 mg/dl	30	0	24	0
Albumin <3.5 g/dl	65	0	55	0
Serum beta2-microglobulin >3.5 mg/l	94	0	54	0
LDH >300 U/l	11	0	16	0

Abbreviations: BJ, Bence-Jones; F, female; Hb, hemoglobin; Ig, immunoglobulin; ISS, International Staging System; LDH, lactate dehydrogenase; M, male; MGUS, monoclonal gammopathy of undetermined significance; MM, multiple myeloma; NS, non-secretory.

**Table 2:** Circulating levels of adhesion molecules in all patients and controls

	*MGUS (*N=*47), median (range)*	*SMM (*N=*61), median (range)*	*NDMM (*N=*141), median (range)*	*RMM (*N=*87), median (range)*
L-Selectin (ng/ml)	1187 (262–2039)	1046 (860–1224)	870 (374–2473)	934 (541–2199)
P-Selectin (ng/ml)	159 (21–305)	160 (40–255)	120 (20–366)	118 (36–380)
E-Selectin (ng/ml)	31 (8–78)	29 (8–73)	31 (6–97)	40 (11–100)
ICAM-1 (ng/ml)	226 (130–649)	256 (167–604)	271 (93–894)	336 (109–782)
VCAM-1 (ng/ml)	388 (131–2117)	504 (174–1751)	799 (226–4567)	788 (178–2987)

	*SMM vs MGUS (*P-*value)*	*NDMM vs MGUS (*P-*value)*	*RMM vs MGUS (*P-*value)*	*NDMM vs SMM (*P-*value)*	*RMM vs SMM (*P-*value)*	*RMM vs NDMM (*P-*value)*
L-Selectin (ng/ml)	0.025	<0.001	<0.001	<0.001	0.003	0.017
P-Selectin (ng/ml)	0.995	<0.001	<0.001	<0.001	<0.001	0.957
E-Selectin (ng/ml)	0.731	0.342	0.006	0.216	0.001	0.021
ICAM-1 (ng/ml)	0.003	0.004	<0.001	0.958	0.002	0.001
VCAM-1 (ng/ml)	0.043	<0.001	<0.001	<0.001	<0.001	0.393

Abbreviations: ICAM-1, intercellular adhesion molecule-1; MGUS, monoclonal gammopathy of undetermined significance; NDMM, newly-diagnosed symptomatic myeloma; RMM, relapse multiple myeloma; SMM, asymptomatic/smoldering myeloma; VCAM-1, vascular cell adhesion molecule-1.
